# Supplementary material for: The Systems Biology Research Tool: evolvable open-source software
Source: BMC Syst Biol. 2008 Jun 29;2:55. doi: 10.1186/1752-0509-2-55 (PMC2446383; doi:10.1186/1752-0509-2-55)
Supplement: Additional file 1 — SBRT Archive. An archive of the current version of the Systems Biology Research Tool. [file 1752-0509-2-55-S1.zip › sbrt-1.4.0/doc/developers_guide/api/sbrt/kernel/math/IntervalComparer.Diff.html]

IntervalComparer.Diff


|  |  |  |
| --- | --- | --- |
| **Overview** \| **Package** \| **Class** \| **Use** \| **Tree** \| **Deprecated** \| **Index** \| **Help** | |  |
| **PREV CLASS**   **NEXT CLASS** | **FRAMES**    **NO FRAMES**     **All Classes** |  |
| SUMMARY: NESTED \| FIELD \| CONSTR \| METHOD | DETAIL: FIELD \| CONSTR \| METHOD |  |


---


## sbrt.kernel.math Class IntervalComparer.Diff<V>

```
java.lang.Object
  sbrt.kernel.math.IntervalComparer.Diff<V>
```

**Type Parameters:**: `V` - the variable type.

**Enclosing class:**: IntervalComparer

---

``` public static class IntervalComparer.Diff<V> extends java.lang.Object ```

Instances of this class are used to hold a variable and a pair of
corresponding intervals.

**Author:**
:   This class was written and documented by Jeremiah Wright while in
    the Wagner lab.

---

| **Method Summary** | |
| --- | --- |
| `Interval` | `getInterval1()` |
| `Interval` | `getInterval2()` |
| `V` | `getVariable()` |

**Methods inherited from class java.lang.Object**

`clone, equals, finalize, getClass, hashCode, notify, notifyAll, toString, wait, wait, wait`

**Method Detail**

### getVariable

```
public V getVariable()
```

---


### getInterval1

```
public Interval getInterval1()
```

---


### getInterval2

```
public Interval getInterval2()
```


---


|  |  |  |
| --- | --- | --- |
| **Overview** \| **Package** \| **Class** \| **Use** \| **Tree** \| **Deprecated** \| **Index** \| **Help** | |  |
| **PREV CLASS**   **NEXT CLASS** | **FRAMES**    **NO FRAMES**     **All Classes** |  |
| SUMMARY: NESTED \| FIELD \| CONSTR \| METHOD | DETAIL: FIELD \| CONSTR \| METHOD |  |


---
